# Endometrial whole metabolome profile at the receptive phase: influence of Mediterranean Diet and infertility

**DOI:** 10.3389/fendo.2023.1120988

**Published:** 2023-04-19

**Authors:** Nerea M. Molina, Lucas Jurado-Fasoli, Alberto Sola-Leyva, Raquel Sevilla-Lorente, Analuce Canha-Gouveia, Susana Ruiz-Durán, Juan Fontes, Concepción M. Aguilera, Signe Altmäe

**Affiliations:** ^1^ Department of Biochemistry and Molecular Biology I, Faculty of Sciences, Granada, Spain; ^2^ Instituto de Investigación Biosanitaria ibs.GRANADA, Granada, Spain; ^3^ Department of Physical Education and Sports, Faculty of Sport Sciences, PROmoting FITness and Health through Physical Activity Research Group (PROFITH), Sport and Health University Research Institute (iMUDS), University of Granada, Granada, Spain; ^4^ Department of Physiology, Faculty of Medicine, University of Granada, Granada, Spain; ^5^ Department of Physiology, Faculty of Pharmacy, University of Granada, Granada, Spain; ^6^ Department of Physiology, Faculty of Veterinary, University of Murcia, Campus Mare Nostrum, Murcia, Spain; ^7^ Institute for Biomedical Research of Murcia IMIB-Arrixaca, Murcia, Spain; ^8^ Unidad de Reproducción, UGC de Obstetricia y Ginecología, Hospital Universitario Virgen de las Nieves, Granada, Spain; ^9^ Department of Biochemistry and Molecular Biology II, Faculty of Pharmacy, University of Granada, Granada, Spain; ^10^ ”José Mataix Verdú” Institute of Nutrition and Food Technology (INYTA), Biomedical Research Centre (CIBM), University of Granada, Granada, Spain; ^11^ CIBER Fisiopatología de la Obesidad y la Nutrición (CIBEROBN), Instituto de Salud Carlos III, Madrid, Spain; ^12^ Division of Obstetrics and Gynecology, Department of Clinical Science, Intervention and Technology, Karolinska Institutet, Stockholm, Sweden

**Keywords:** endometriosis, endometrium, folic acid, lifestyle, metabolomics, Mediterranean diet, recurrent implantation failure, unexplained infertility

## Abstract

**Introduction:**

Several metabolite classes have been identified in human endometrium, including lipids, nucleotides, amino acids, organic acids, and sugars. The first studies suggest the importance of metabolites in endometrial functions, as imbalance in uterine metabolites has been associated with low implantation rate and endometriosis. Nevertheless, most of studies have put emphasis on specific metabolite classes, and we lack the knowledge of the whole metabolome composition in human uterus. Further, a healthy dietary pattern has been shown to potentially protect against different endometrial dysfunctions and is a potential modulator of metabolomic composition and, consequently, the intrauterine microenvironment. The Mediterranean Diet (MD), characterized by a high intake of fruits, vegetables, cereals, nuts, legumes, fish, and olive oil, and a low consumption of meat, dairy products, and processed foods, has been associated with a wide range of benefits for health. Indeed, the MD pattern has displayed a beneficial role in endometriosis management and fertility; however, the relationship between the MD and the endometrial metabolome is still unknown. In our study, we set out to analyze receptive-phase endometrial metabolome profiles among women with infertility and their associations with MD.

**Methods:**

The study included women with male factor infertility (n=8), unexplained infertility (n=10), recurrent implantation failure (n=14), and endometriosis (n=13). The endometrial metabolome was analyzed with ultrahigh-performance liquid chromatography-tandem mass spectroscopy (UPLC–MS/MS). The MD adherence of the participants was assessed using the 14-point MEDAS questionnaire of adherence to the MD.

**Results:**

We provide the whole metabolome profile of the endometrium, where 925 different metabolites were identified. Among these metabolites, lipids comprised the largest part, where polyunsaturated fatty acids (PUFAs) prevailed. Women with endometriosis and recurrent implantation failure were found to have lower levels of PUFAs compared to women with male factor and unexplained infertility (i.e., no clear endometrial alterations), identifying a metabolome profile associated with infertility diagnoses where altered endometrial functions are suspected. Moreover, MD adherence seemed to be associated with the endometrial metabolomic profile in a manner dependent on the health status of the uterus.

**Conclusion:**

The study findings provide insight into the molecular background of female infertility and lead to identification of potential molecular biomarkers and possibilities for modulating the endometrial microenvironment and, thereby, endometrial functions involved in embryo implantation and infertility.

## 1 Introduction

The human endometrium is a dynamic tissue that undergoes continuous cycles of shed, repair, regeneration, and remodeling to prepare for the embryo implantation (1). Due to the complexity of the involved mechanisms and the relative lack of information, embryo implantation process in humans remains the “black box” (2). Unfavorable endometrial microenvironment could lead to endometrial dysfunction promoting different disorders including embryo implantation failure and infertility, abnormal uterine bleeding, endometriosis, and endometrial cancer among others (1, 3). In this sense, the novel “omics” techniques based on the massive molecular characterization of DNA, RNA, proteins, and other small molecules synthesized have allowed to define the molecular phenotype of the receptive-phase endometrium at the mid-secretory phase (so-called “window of implantation”) and the genomic, epigenomic, proteomic, and metabolomic methods have helped to gain insight into the molecular markers of the mid-secretory endometrial functions in health and disease (3–8).

Metabolomics has emerged as a powerful tool to identify small molecules with a molecular mass below 1200Da that participate as intermediates and final downstream products of cellular processes (i.e., metabolites), which contribute to a close real-time understanding of the functional phenotype (9). Despite the growing number of metabolomic studies, the use of the whole metabolite profile analysis related to uterine health is still scarce. The first endometrial metabolome studies have analyzed women with endometriosis and have suggested several potential biomarkers of the disease (10–12). The whole metabolome profiling in the uterus of fertile and infertile women undergoing assisted reproductive techniques showed that specific metabolomic signatures contribute to beneficial endometrial microenvironment to achieve pregnancy (13–15). These are the first findings indicating that metabolomics is a powerful tool in unravelling uterine health and understanding receptive-phase endometrial functions to achieve successful embryo implantation.

Factors that may influence uterine microenvironment include nutrition together with other lifestyle factors. It has been previously shown that a healthy dietary pattern might protect against different endometrial dysfunctions such as infertility, endometriosis, polycystic ovary syndrome, dysmenorrhea, or endometrial cancer (16, 17). In fact, food groups, such as fruits and vegetables, could promote fertility; while other dietary elements, including trans-fatty acids, alcohol, and caffeine, have been associated with adverse effects on fertility (18). The Mediterranean Diet (MD), characterized by a high consumption of fruits, vegetables, cereals, nuts, legumes, fish, and olive oil, and a low consumption of meat, dairy products, and processed foods, has been associated with a wide range of benefits for health (19). Indeed, the MD has been shown to have a beneficial role in endometriosis management (20), fertility (21, 22), and polycystic ovary syndrome (23). However, although lifestyle interventions have shown to influence the uterine proteome (24), the relationship between the MD and the endometrial metabolome is still unknown. In the current study, we set out to analyze receptive-phase endometrial metabolome profiles among women with infertility and their associations with the MD.

## 2 Materials and methods

### 2.1 Study subjects and design

This cross-sectional study was approved by the Ethics Committee of the Junta de Andalucía (CEIM/CEI 0463-M1-18r). All procedures carried out in the present study were in accordance with the last revised ethical guidelines of the Declaration of Helsinki and the legally enforced Spanish regulation, which regulates the clinical investigation of human beings (RD 223/04). Written informed consent was obtained from all subjects prior to inclusion.

Forty-five women of infertile couples were recruited at the Reproductive Unit (Virgen de las Nieves University Hospital, Granada) between March 2019 and April 2021. All participants attended the Reproductive Unit after at least 1 year of unprotected sex and not being able to conceive. The inclusion criteria involved women entering an infertility treatment. Infertility causes comprised endometriosis, recurrent implantation failure (RIF), unexplained infertility, or male factor infertility. Endometriosis was diagnosed by laparotomy or laparoscopic surgery and histological confirmation, and RIF was defined as repeated implantation failure after the transfer of 3 good-quality embryos. Unexplained infertility was considered when the medical examination yielded no information about reproductive complications in the couple. Lastly, male factor was defined considering the reference values of the World Health Organization manual for semen analysis (25). Patients with age ≥43 years old, with hormone therapy, gynecological tumors, systemic diseases, pelvic inflammatory disease, or other pelvic pathological conditions were excluded from the study.

Participants were invited to complete an extensive lifestyle questionnaire to collect information including demographic characteristics. Body mass index (BMI) was calculated from the measured weight and height data. Ovulation day was estimated using a digital ovulation test (ClearBlue, Swiss Precision Diagnostics GmbH, Geneva, Switzerland) to precisely schedule the endometrial biopsy collection 7-9 days after the luteinizing hormone (LH) peak (day LH+7-9, window of implantation). After insertion of the speculum, the endometrial tissue sample was collected using the endometrial curette device (Gynétics, Medical Products, Hamont-Achel, Belgium). All biopsies were placed in cryovials, were snap-frozen in the gas phase of liquid nitrogen and stored at -80°C until analysis.

### 2.2 Endometrial metabolomic profiling

The untargeted metabolomics of endometrial samples were analyzed at Metabolon Inc. (Morrisville, NC, USA) on a platform consisting of four independent ultrahigh-performance liquid chromatography-tandem mass spectroscopy (UPLC–MS/MS) instruments as previously described (26). Briefly, samples were prepared using the automated MicroLab STAR^®^ system from the Hamilton Company. Several recovery standards were added prior to the first step in the extraction process for quality-control (QC) purposes. To recover chemically diverse metabolites, proteins and other macromolecules were precipitated with methanol under vigorous shaking for 2 min (Glen Mills GenoGrinder 2000) followed by centrifugation. The resulting supernatant was divided into 4 aliquots, then placed briefly in a TurboVap (Zymark, Hopkinton, MA, USA) to remove the organic solvent and stored overnight under nitrogen before preparation for analysis. The sample extract was then reconstituted in solvents compatible with each of the four methods. Thus, each aliquot was run using a different method: 1) acidic positive-ion conditions chromatographically optimized for more hydrophilic compounds (reverse phase [RP]/UPLC-MS/MS Pos Early); 2) acidic positive-ion conditions chromatographically optimized for more hydrophobic compounds (RP/UPLC-MS/MS Pos Late); 3) basic negative-ion-optimized conditions (RP/UPLC-MS/MS Neg). These methods used separate acid- and base-dedicated C18 columns (Waters UPLC BEH C18, 2.1×100 mm, 1.7 μm). The fourth aliquot was analyzed via negative ionization after elution from a hydrophilic interaction liquid chromatographic (HILIC) column (Waters UPLC BEH Amide, 2.1x150 mm, 1.7 µm) (HILIC/UPLC-MS/MS Polar). The details of the used solvents and chromatography are described before (27). All UPLC-MS/MS methods used a Waters ACQUITY UPLC (Waters, Milford, MA, USA) coupled to a Thermo Scientific Q-Exactive high resolution/accurate mass spectrometer (Thermo Fischer, Waltham, MA, USA) equipped with a heated electrospray ionization (HESI-II) source and Orbitrap mass analyzer. The MS analysis alternated between MS and data-dependent MS^n^ scans using dynamic exclusion. The MS scan range varied slightly between methods but covered 70-1000 m/z and operated at 35,000 mass resolution.

Three types of controls were analyzed along with the experimental samples: 1) a pooled sample generated from a small volume of each experimental sample (i.e., technical replicate); 2) extracted water (i.e., blank); and 3) a cocktail of QC standards spiked into every analyzed sample for instrument performance monitoring. Instrument variability was determined by calculating the median relative standard deviation (RSD) for the internal standards that were added to each sample prior to injection into the mass spectrometers (median RSDs = 3%). Overall process variability was determined by calculating the median RSD for all endogenous metabolites (i.e., non-instrument standards) present in 100% of the pooled matrix samples, which were technical replicates of pooled samples (median RSD = 7%). Experimental samples and controls were randomized across the platform run.

### 2.3 Endometrial metabolome detection and identification

Raw data, peak-identified, and QC was extracted and processed using Metabolon’s hardware and software as described previously (28). Endometrial metabolites were identified by comparison of the ion features in the experimental samples to reference libraries of authenticated standards with known chromatographic retention index (RI), mass to charge ratios (m/z), and MS/MS spectral fragmentation signatures. Further, biochemical identification was based on 3 criteria: RI within a narrow RI window (150 RI units [≈10s]), accurate mass match ( ± 10ppm), and quality of MS/MS fragmentation match. While there may be similarities between these molecules based on one of these factors, the use of all three data points allows to distinguish and differentiate biochemicals. To ensure high quality of the dataset, QC and curation processes were subsequently used to confirm accurate and consistent chemical assignment and remove system artefacts and background noise. Peaks were quantified through area-under-the-curve analysis and these peak area values allowed the determination of relative quantification among samples (29).

Some compounds were detected by multiple methods (i.e., RP/UPLC-MS/MS Pos Early, RP/UPLC-MS/MS Pos Late, RP/UPLC-MS/MS Neg, or HILIC/UPLC-MS/MS Polar). The UPLC–MS/MS method used to evaluate each compound was pre-selected based on sensitivity and reproducibility criteria (“Platform” column of **Supplementary Table S1**).

### 2.4 Adherence to Mediterranean Diet

Adherence to the MD was assessed using the 14-point MEDAS questionnaire of adherence to the MD as previously reported and validated (30). The MEDAS questionnaire includes 12 questions related to frequency intake of key foods, and 2 questions related to specific dietary habits of the MD (**Supplementary Table S2**). Each question scores 0 or 1 point. The global score ranges from 0 to 14, being 0 points null adherence and 14 points complete adherence to the MD. Then, participants were categorized in high adherence (≥9 points) or low adherence (<9 points) to the MD, following previous categorization (30). The consumption of folic acid supplement was also registered among the participants with an additional question together with the MEDAS questionnaire (**Supplementary Table S2**).

### 2.5 Statistical analyses

The normal distribution assumption was tested using the Shapiro-Wilk test, visual histograms, and Q-Q plots. Non-normally distributed variables (i.e., endometrial metabolomic signature) were log_10_-transformed before further analysis. The baseline characteristics and outcomes of the study participants were expressed as mean ± standard deviation (unless otherwise stated). Differences in demographic characteristics between the subjects of our study were analyzed using independent sample *t*-test or chi-square tests where appropriate.

Multivariate statistical analyses (i.e., partial least squares discriminant analysis [PLS-DA]) were performed on the entire metabolomics dataset using the MetaboAnalyst 5.0 (https://www.metaboanalyst.ca) to investigate potential difference in the metabolomic signature between women with low and high adherence to MD, different infertility diagnoses, and between women supplemented with folic acid and women with no supplementation. To increase the importance of low abundance ions without significant amplification of noise, raw data were normalized by Pareto scaling (31).

To find further differences in metabolite composition between the different groups, one-way analysis of covariance (ANCOVA) controlling for age and BMI was applied. Besides, we conducted Pearson partial correlation analyses to examine the relationship between the adherence to the MD and the endometrial metabolomic signature adjusting for age and BMI. The correlation analyses were performed pooling all participants together and splitting the cohort based on the results of the uterine examination (see results section).

Normality and descriptive analyses were performed in Statistical Package for the Social Sciences (SPSS 28.0, IBM Corp, Armonk, NY, USA). All statistical and correlation analyses were performed using the R software (V.3.6.0; R Foundation for Statistical Computing). Whisker’s plot and volcano plots were built using GraphPad Prism software v.9 (GraphPad Software, San Diego, CA, USA). All *p*-values were corrected by controlling for the multiple testing, False Discovery Rate (FDR) (32). Statistical significance was set *p*-value<0.05 after FDR correction.

## 3 Results

### 3.1 Study subjects

The study cohort included a total of 45 women with infertility. The characteristics of the participants are presented in **Table 1**. Participants were primarily Caucasian, with just one Hispanic woman.

**Table 1:** Patient’s demographics based on the adherence to Mediterranean Diet (MD).

	Total (n=45)	High adherence to the MD (n=25)	Low adherence to the MD (n=20)	*p*-value^a^
Age (years)	34.62 ± 3.67	34.56 ± 3.82	34.7 ± 3.57	0.901
BMI (kg/m^2^)	24.62 ± 4.19	25.13 ± 4.62	23.99 ± 3.6	0.368
Infertility diagnosis, n (%)				
Male factor	8 (18)	3 (12)	5 (25)	0.3
Unexplained infertility	10 (22)	8 (32)	2 (10)	
Recurrent implantation failure	14 (31)	7 (28)	7 (35)	
Endometriosis	13 (29)	7 (28)	6 (30)	
Uterine examination, n (%)				
No endometrial-factor infertility (NEFI)	18 (40)	11 (44)	7 (35)	0.54
Endometrial-factor infertility (EFI)	27 (60)	14 (56)	13 (65)	
Folic acid supplementation*, n (%)				
Yes	26 (58)	16 (64)	10 (50)	0.355
No	19 (42)	9 (36)	10 (50)	

Data values are presented as n (%) or mean ± standard deviation for continuous traits.

*The number of women varies due to nonresponse to this item: high adherence to the MD (N=23), low adherence to the MD (N=18).

a t-test (continuous variable) or Pearson’s chi-square test of independence.

BMI, body mass index.

**Figure 1:**
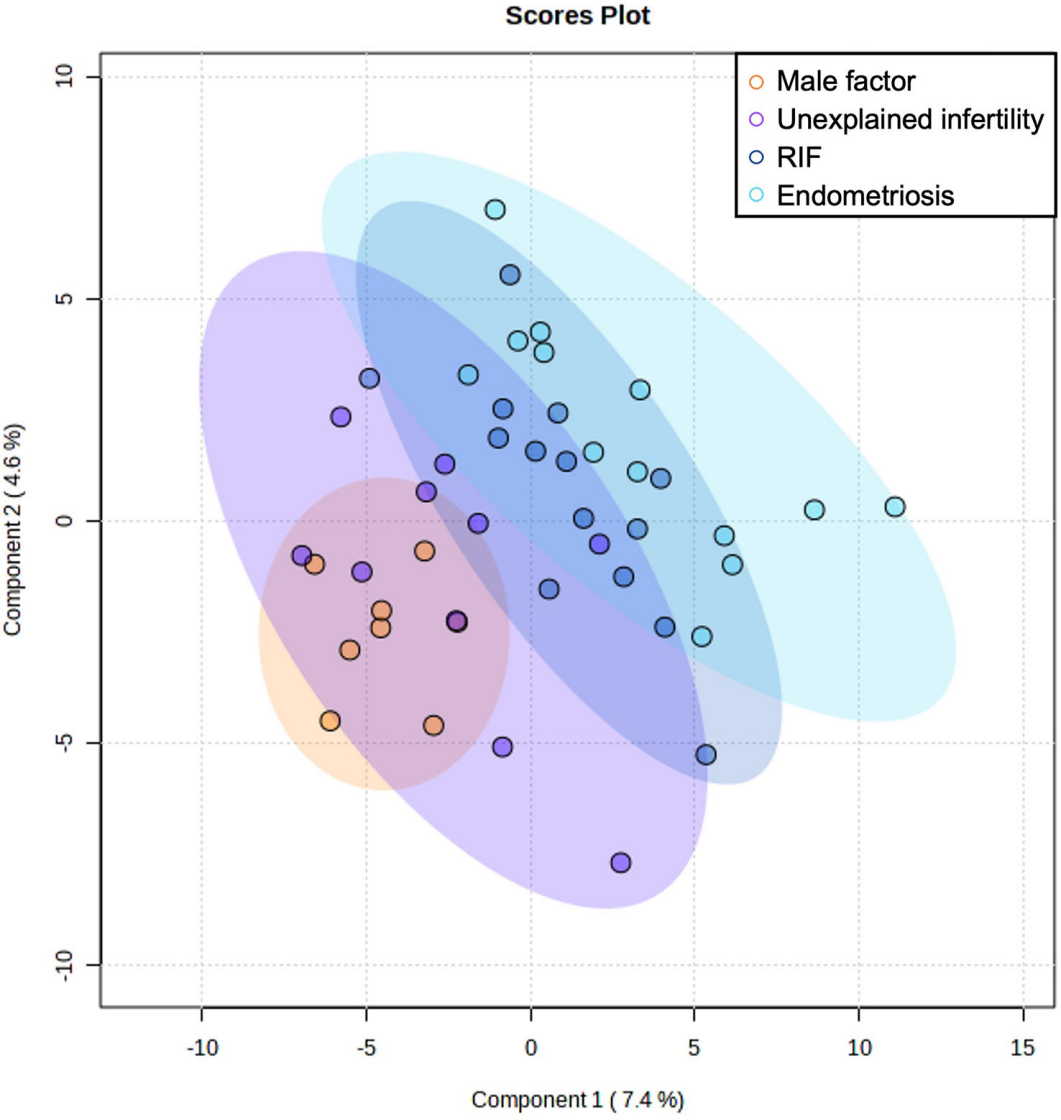
Multivariate Partial Least Squares Discriminant Analysis scores plot from women with male factor infertility, unexplained infertility, endometriosis, and recurrent implantation failure (RIF).

Based on the MEDAS score, the participants were categorized as high adherence (n=25) or low adherence (n=20) to the MD. We also studied the differences in metabolome profiles depending on the infertility diagnosis to relate the uterine microenvironment with endometrial dysregulation. To increase the statistical power of our analysis and based on the thorough clinical examination, 27 patients with endometriosis and RIF were grouped as endometrial-factor infertile (EFI) and 18 women with male factor infertility and unexplained infertility were grouped together as no endometrial-factor infertility (NEFI) was detected. This grouping was further supported by the metabolome signatures detected (**Figure 1**), where women with RIF or endometriosis demonstrated similar metabolite profiles while women with male factor infertility and unexplained infertility grouped together. Statistical tests revealed that the different groups were age- and BMI-matched (**Table 1**).

### 3.2 Metabolome composition

Nine hundred twenty-five metabolites from diverse chemical classes were identified from endometrial tissue samples using an untargeted metabolomic approach. These 925 metabolites included amino acids, lipids, nucleotides, carbohydrates, and xenobiotics among others. The list of identified compounds is provided in **Supplementary Table S1**. The most abundant metabolites in the entire cohort were N1, N12-diacetylspermine, adenosine 5’-monophosphate (AMP), 4-ethylphenylsulfate, guanosine 5’-monophosphate (5’-GMP), 2-hydroxy-3-methylvalerate, and UDP- glucuronate.

#### 3.2.1 Metabolite profiles and MD adherence

**Figure 2:**
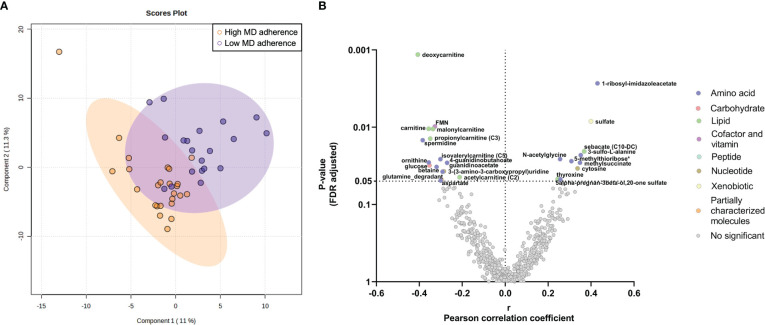
Endometrial metabolomic signature analysis in women with high and low adherence to Mediterranean Diet (MD). **(A)** Multivariate Partial Least Squares Discriminant Analysis scores plot from women with high and low MD adherence. **(B)** Partial correlation analyses between the MD global score and the endometrial metabolome. The X-axis represents Pearson partial correlations coefficients, whereas the Y-axis represents the FDR-adjusted *p*-values of the correlations. Grey dots represent non-significant correlations, whereas colored dots represent statistically significant correlations (*p*-value<0.05 after FDR correction).

When we compared metabolome based on adherence to diet, the PLS-DA scores plot did not show any different metabolomic signature between the study groups with high and low adherence to the MD (**Figure 2A**) and no significant differences were detected after correction between the metabolites and the different groups (**Supplementary Table S3**).

When focusing on metabolite classes, the adherence to the MD showed statistically significant correlations with amino acids and lipids levels (all *p*-values<0.05 after FDR correction; **Figure 2B**). Specifically, the adherence to the MD was positively correlated with amino acids related to the histidine metabolism (1-methyl-5-imidazoleacetate and 1-ribosyl-imidazoleacetate), to the methionine, cysteine, SAM, and taurine metabolism (N-acetylmethionine sulfoxide, 5-methylthioribose, and 3-sulfo-L-alanine), and the thyroxine amino acid (**Figure 2B**, **Supplementary Table S4**). In contrast, we observed that the adherence to the MD was negatively correlated with different amino acid metabolites (argininate, ornithine, betaine, spermidine, and aspartate) and with carnitines (e.g., deoxycarnitine, carnitine, malonylcarnitine, and 2-methylhexanoylcarnitine) (**Figure 2B**, **Supplementary Table S4**).

#### 3.2.2 Metabolite profiles and endometrial-factor infertility

**Figure 3:**
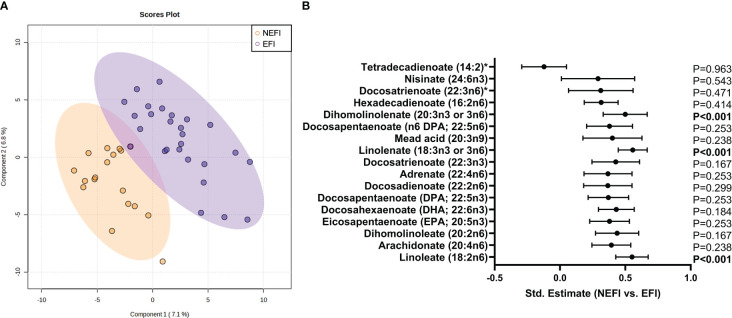
Endometrial metabolomic signature analysis in women with no detectable endometrial dysfunction in comparison to patient group with endometriosis and recurrent implantation failure. **(A)** Multivariate Partial Least Squares Discriminant Analysis scores plot from women with no detectable endometrial dysfunction and women with endometrial-factor infertility. **(B)** Statistical differences in long chain polyunsaturated fatty acid metabolism between women with no detectable endometrial dysfunction and women with endometrial-factor infertility. Statistical significance was set *p*-value<0.05 after FDR correction. NEFI, no endometrial-factor infertility; EFI, endometrial-factor infertility.

Comparing groups based on the uterine examination, the PLS-DA scores plot did not discriminate the metabolomic signature between patients with EFI and second group with NEFI (**Figure 3A**). However, dihomolinolenate (20:3 n-3 or n-6), linolenate (18:3 n-3 or n-6), and linoleate (18:2 n-6) metabolites related with the polyunsaturated fatty acids (PUFAs) pathway were significantly increased in NEFI women compared to EFI women (all *p*-values<0.001 after FDR correction; **Figure 3B**, **Supplementary Table S5**).

**Figure 4:**
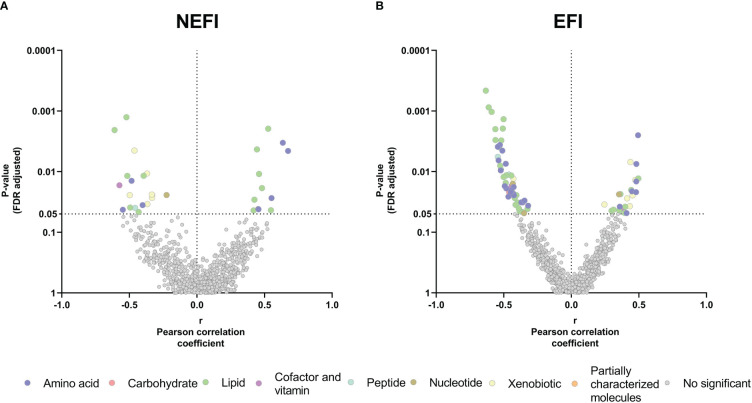
Relationship between the Mediterranean Diet adherence and the endometrial metabolome in **(A)** women with no detectable endometrial dysfunction and **(B)** patient group with endometriosis and recurrent implantation failure. The X-axis represents Pearson partial correlations coefficients, whereas the Y-axis represents the FDR-adjusted *p***-**values of the correlations. Grey dots represent non-significant correlations, whereas colored dots represent statistically significant correlations (*p*-value<0.05 after FDR correction). NEFI, no endometrial-factor infertility; EFI, endometrial-factor infertility.

Next, we performed the correlation analyses in parallel among patient group of women with EFI and among group of women with NEFI and we observed a higher number of significant correlations between the adherence to the MD and metabolites in women with EFI than in women with no detectable endometrial dysfunction (**Figure 4**). In the NEFI group, up to 31 metabolites correlated to MD. On one hand, the adherence to the MD was positively correlated to different fatty acids (2S,3R-dihydroxybutyrate, 2-hydroxystearate, and 2-hydroxypalmitate), to progestin steroids (pregnanediol-3-glucuronide, and 5alpha-pregnan-3beta,20beta-diol monosulfate) while was negatively correlated to metabolites involved in the xanthine metabolism (1,3,7-trimethylurate, 5-acetylamino-6-amino-3-methyluracil, caffeine, 1,7-dimethylurate, and 1-methylurate) (**Figure 4A**, **Supplementary Table S4**). Contrary, in patients with EFI, a total of 73 metabolites correlated to MD. We observed a positive correlation between the adherence to the MD and metabolites involved in the xanthine metabolism (i.e., 5-acetylamino-6-amino-3-methyluracil, 1,3,7-trimethylurate, 1-methylxanthine, and paraxanthine) and in the metabolism of ceramides (i.e., dihydroceramides, hexosylceramides, and lactosylceramides) (**Figure 4B**, **Supplementary Table S4**). We also found in women with EFI, a negative correlation between the adherence to the MD and carnitine metabolites (e.g., carnitine, deoxycarnitine, or acyl-carnitines), primary bile acid metabolites (i.e., cholate, glycochenodeoxycholate 3-sulfate, glycochenodeoxycholate, and chenodeoxycholate), and different amino acid metabolites (e.g., aspartate, argininate, ornithine, orotidie, spermidine, betaine, or homocysteine) (**Figure 4B**, **Supplementary Table S4**).

#### 3.2.3 Metabolite profiles and combination of MD adherence and endometrial factor

Additionally, we combined the 2 classification features (i.e., MD adherence and endometrial-factor infertility) for comparisons and 4 groups were established: high MD adherence and NEFI (n=11), high MD adherence and EFI (n=14), low MD adherence and NEFI (n=7), and low MD adherence and EFI (n=13) (**Table 1**). When we compared metabolome profiles among these groups, linolenate metabolite was significantly more present among women with high MD adherence and NEFI compared to women with low MD adherence and EFI (*p*-value=0.014 after FDR correction, **Supplementary Figure S1**).

#### 3.2.4 Metabolite profiles and folic acid intake

**Figure 5:**
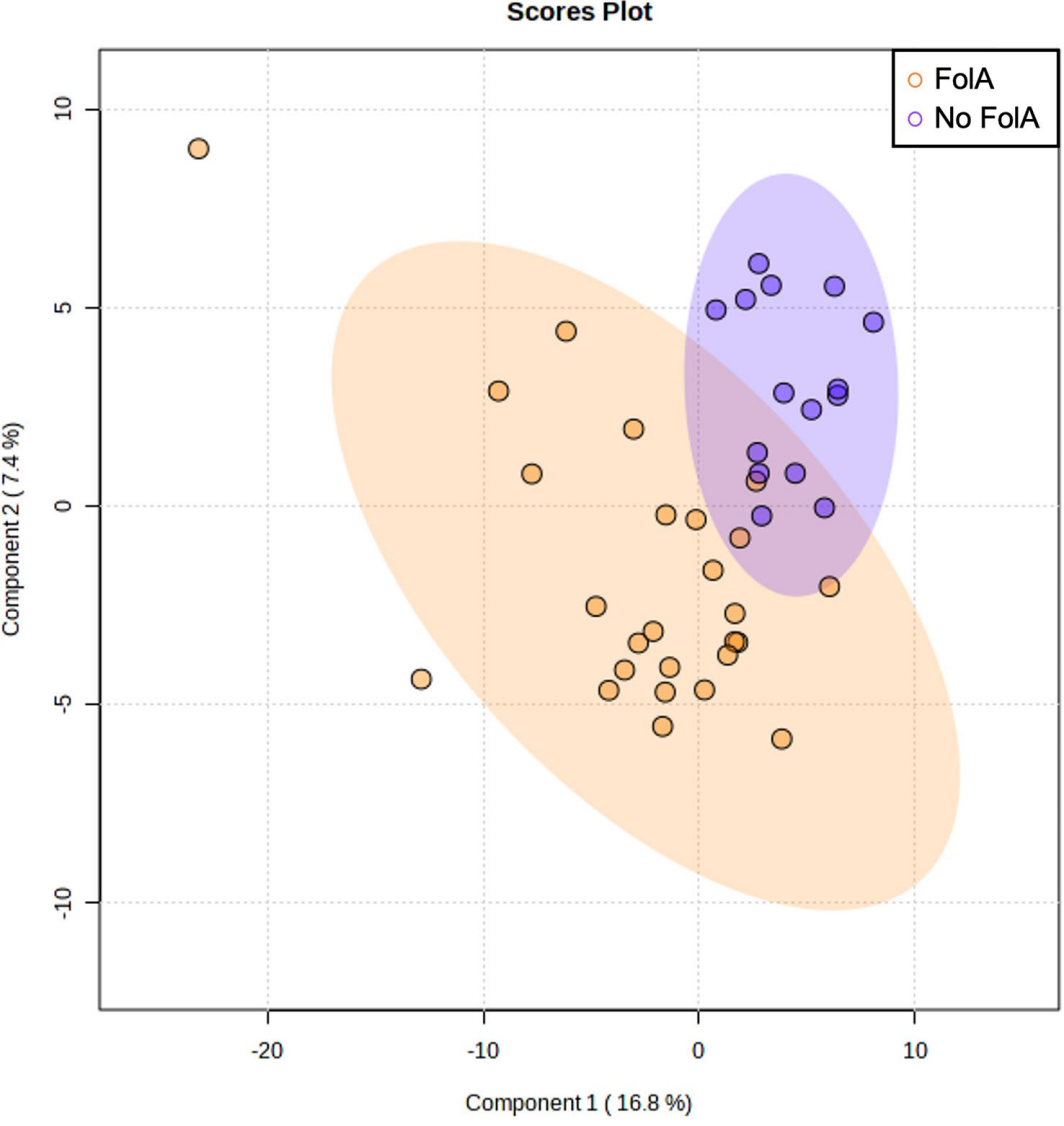
Multivariate Partial Least Squares Discriminant Analysis scores plot from women supplemented with folic acid (FolA) and women with no supplementation.

Regarding the folic acid supplementation, the PLS-DA scores plot did not show any different clusters (**Figure 5**) nor were significant differences observed in the metabolomic profile when comparing the groups of women who were supplemented with folic acid or not (**Supplementary Table S6**).

## 4 Discussion

In this cross-sectional study, we used an untargeted metabolomics approach to profile the endometrial metabolome and to assess possible differences in the metabolite composition between women with low and high adherence to MD, different infertility diagnoses (i.e., male factor infertility, unexplained infertility, RIF, and endometriosis), and taking also into account folic acid supplementation. We provide the whole metabolome profile of the endometrium, where 925 different metabolites were identified. Among these metabolites, lipids comprised the largest percentage (40%), where PUFAs such as mead acid, dihomo-linolenate, and docosatrienoate prevailed. Our main results indorse those women with endometrium-related infertility (i.e., endometriosis or RIF) have lower levels of PUFAs in the endometrium compared to women with no clear endometrial factor infertility (i.e., male factor and unexplained infertility). Further, supporting our grouping based on infertility diagnosis, male factor and unexplained infertility demonstrated closer metabolome patterns while RIF and endometriosis were more similar. Moreover, adherence to MD seemed to be associated with the endometrial metabolomic profile, differing between women with distinct infertility diagnoses. In this way, adherence to MD could be related to the levels of different fatty acids, progestin steroids, xanthine metabolites, carnitines, amino acids, and bile acids in a manner dependent on the health status of the uterus.

In our study, PUFAs prevailed in all the women studied. When comparing the study groups based on the uterine examination, dihomolinolenate, linoleate, and linolenate were detected at lower levels in the endometrium of women with RIF or endometriosis. PUFAs are fatty acids with multiple double bonds in their structures which cannot be synthesized by the human body, being considered essential fatty acids (33). Linoleate and linolenate are the conjugated acids of linoleic acid (LA) and α-linolenic acid (ALA), respectively, which are the primary PUFAs in Western diets (34). PUFAs are shown to play a substantial role in the regulation of body homeostasis and are considered crucial for reproductive health (35). These lipids participate in female fertility at different reproductive phases, including oocyte maturation and quality, and embryo implantation (36–39). Our study results indicate that microenvironment in the uteri of women with RIF or endometriosis could be lacking in PUFAs, which could negatively impact the endometrial functions.

We detected low levels of linolenate in women with EFI compared to women with NEFI. Interestingly, when we combined MD adherence and endometrial-factor infertility features, the results did support that linolenate was less present in the endometrium of women with low MD adherence and EFI compared to women with high MD adherence and NEFI. Previous studies show contradicting results, as negative correlations between ALA levels in different biological fluids (i.e., serum, follicular fluid) of women undergoing infertility treatment and pregnancy rates (40) and number of metaphase II oocytes (41) have been found. Similarly, serum ALA levels have shown positive association with the presence of endometriosis (40). These discrepancies could be due to the different analyzed specimens (serum *versus* endometrial biopsy). Some evidence also manifests that LA has negative effects on oocyte maturation (33), which could be mediated by the role of lipids in the physical properties and biological functions of membranes (13). Other authors measured the LA : ALA ratio (n-6 to n-3) and they found that women with higher LA : ALA ratios had greater embryo implantation and pregnancy rates compared to women with reduced LA : ALA ratios (42, 43), suggesting a potential role for increased LA to enhance endometrial inflammation and receptivity (43) and for elevated ALA to disturb embryo implantation (35). It is worth mentioning that LA is a precursor to docosapentaenoic acid (n-6). Thus, our data seem to support the previous results as we found that the docosapentaenoate (i.e., conjugated acid of docosapentaenoic acid) is less present in the endometrium of women with EFI. Nevertheless, another study revealed that docosapentaenoic acid was higher in women with no implantation compared to women with successful implantation (44). In this way, further investigation is required to unravel the role of n-6 and n-3 PUFAs in endometrial receptivity as this ratio imbalance could lead to aberrant endometrial functions.

Likewise, we observed a positive correlation between adherence to MD and metabolites involved in the metabolism of ceramides in women with EFI. In line, we noticed that ceramide metabolites were higher in women with no endometrial dysfunction. Although with no statistical significance, these results agree with a previous study where ceramides were detected at lower amount in the endometrial fluid of women with endometriosis compared to women without endometriosis (10).

Another family of metabolites that we found dysregulated in women with endometrium-related infertility were progestin steroids (i.e., intermediates of progesterone metabolism) which are shown to contribute to fertility and the maintenance of pregnancy (45). We revealed that lower adherence to MD was related to lower endometrial levels of these hormonal intermediates in women with endometrium-related infertility. A recent work demonstrated that premature rise in the serum progesterone level in the late follicular phase led to a disrupted endometrial lipid profile in the endometrium during the peri-implantation period (44), suggesting that the altered lipid pattern could desynchronize endometrial receptivity and early embryo implantation. There seems to be a link between progesterone metabolism and lipid profiles, which needs further investigation.

Additional interesting result of our study is that a higher adherence to MD correlated with lower levels of carnitine metabolites within the whole cohort. Accordingly, the patient group of women with EFI exhibited a negative correlation between MD adherence and acylcarnitines. It could be that MD confers protection against endometriosis since increased carnitine levels were observed in women with endometriosis (10) and correlated with the cytokine and cellular profile of endometriosis (46). Moreover, high levels of acylcarnitines have also been associated with beta-oxidation dysfunction, participating in inflammation processes (47). Further, we detected a negative correlation of MD adherence with bile acid metabolites in women with EFI, which corroborates that high levels of bile acids are a negative factor for health (48). All these results related to lipid metabolism endorse that lipidome is an essential aspect of the complex process of endometrium receptivity and embryo implantation (35) and alterations in lipid metabolism could lead to endometrial dysfunctions.

We also found a negative correlation between the MD adherence and xanthine metabolites (i.e., products of the purine and xenobiotics degradation) in women with NEFI and a positive correlation in women with EFI. A healthy diet in women without endometrial abnormal functioning has been associated with lower levels of these metabolites, which were found to be elevated in previous studies in women with endometrial disorders (e.g., caffeine (49), xanthosine (11)). However, studies involving xanthines in female reproduction have been mainly focused on their use in oocyte *in vitro* maturation (49) and other reproductive outcomes need to be considered.

Some aspects of the current study should be considered as limitations. Firstly, the cross-sectional design does not allow the establishment of causality. Secondly, our results should be interpreted as preliminary due to the limited sample size, which might have underpowered the detection of statistical differences in metabolite composition between the studied groups. Further studies investigating a MD intervention on the endometrial metabolomic signature are thus required to confirm our results.

Despite these shortcomings, the strength of our study is the focus on a very narrow time-frame of the cycle, the mid-secretory phase, and all the samples were collected at the day LH+7, minimizing thereby the heterogeneity and it enabled us to study the receptive-phase uterine microenvironment. Further, existing metabolomic studies of female reproductive health have mainly been performed in serum samples and assessed a preestablished groups of metabolites, while in our study we analyzed for the first time the endometrial whole metabolic signature with an untargeted method.

This study presents the endometrial whole metabolome profile, demonstrating that PUFAs are prevailing in the human uterus. In conclusion, we showed that women with RIF or endometriosis displayed lower levels of PUFAs in the endometrium compared to women with no clear endometrial alterations, identifying a metabolomic profile associated with infertility diagnoses where altered endometrial functions are suspected (i.e., endometriosis and RIF). In addition, adherence to the MD was associated with the endometrial metabolic profile in a manner dependent on the health status of the uterus. Our study findings help to understand the molecular background of female infertility, which could allow to identify potential molecular biomarkers and take personalized medicine into the reproductive clinical practices. With a healthy diet, endometrial microenvironment could be changed from unfavorable to favorable, which could have an influence on endometrial functions.

## Data availability statement

The original contributions presented in the study are included in the article/**Supplementary Materials**. Further inquiries can be directed to the corresponding authors.

## Ethics statement

The studies involving human participants were reviewed and approved by Ethics Committee of the Junta de Andalucía (CEIM/CEI 0463-M1-18r). The patients/participants provided their written informed consent to participate in this study.

## Author contributions

NM, LJ-F, AS-L, RS-L, and SA conceived and designed the study. NM, AS-L, JF, and SR-D recruited the participants and obtained the samples. LJ-F and RS-L processed the diet data. NM and LJ-F analyzed the data and drafted the manuscript. AS-L, RS-L, AC-G, JF, SR-D, CA, and SA revised the manuscript. All authors contributed to the article and approved the submitted version.

## Supplementary material

Supplementary Table 1 Dataset comprising the average peak intensity and detection platform of each identified metabolite in women with high and low adherence to the Mediterranean Diet.

Supplementary Table 2 Quantitative score (14-item) of adherence to the Mediterranean Diet. The participants were asked to choose the best option for each question. ‘<’ means less than. The option marked with grey color is the one used as criteria for 1 point. The participants also self-reported their folic acid intake.

Supplementary Table 3 Statistical differences in metabolites between groups based on Mediterranean Diet (MD) adherence (i.e., high and low MD adherence).

Supplementary Table 4 Correlation analyses between the Mediterranean Diet global score and the endometrial metabolome.

Supplementary Table 5 Statistical differences in metabolites between groups based on uterine examination (i.e., women with no endometrial-factor infertility and patients with endometrial-factor infertility).

Supplementary Table 6 Statistical differences in metabolites between groups based on folic acid supplementation.

Supplementary Figure 1 Statistical difference in linolenate metabolite between women with high Mediterranean Diet (MD) adherence and no endometrial-factor infertility and women with low MD adherence and endometrial-factor infertility. Statistical significance was set p-value<0.05 after FDR correction. EFI, endometrial-factor infertility; NEFI, no endometrial-factor infertility.
