# Supplementary material for: Endometrial whole metabolome profile at the receptive phase: influence of Mediterranean Diet and infertility
Source: Front Endocrinol (Lausanne). 2023 Apr 19;14:1120988. doi: 10.3389/fendo.2023.1120988 (PMC10155813; doi:10.3389/fendo.2023.1120988)
Supplement: Supplementary Table 2 — Quantitative score (14-item) of adherence to the Mediterranean Diet. The participants were asked to choose the best option for each question. ‘<’ means less than. The option marked with grey color is the one used as criteria for 1 point. The participants also self-reported their folic acid intake. [file Table_2.docx]

**Supplementary Table 2.** Quantitative score (14-item) of adherence to the Mediterranean Diet. The participants were asked to choose the best option for each question. '<' means less than. The option marked with grey color is the one used as criteria for 1 point. The participants also self-reported their folic acid intake.

| **Question** | **Frequency** | |
| --- | --- | --- |
| 1. Do you use olive oil as your main cooking fat? | Yes | No |
| 2. How many tablespoons of olive oil do you consume in total per day (including that used for frying, meals away from home, salad, etc.) | <4 | At least 4 |
| 3. How many servings of vegetables do you consume per day? (Garnishes or side dishes = ½ serving, 1 serving= 200 grams) | <2 | At least 2 |
| 4. How many pieces of fruit (including natural juice) do you consume per day? | <3 | At least 3 |
| 5. How many servings of red meat, hamburgers, sausages, or cold meats do you consume per day? (1 serving 100-150 grams) | <1 | At least 1 |
| 6. How many servings of butter, margarine, or cream do you consume per day? (1 single serving 12 grams) | <1 | At least 1 |
| 7. How many carbonated and/or sweetened beverages (2 dl) (soft drinks, colas, tonic, bitters) do you consume per day? | <1 | At least 1 |
| 8. Do you drink wine? How many glasses do you drink per week? | <7 | At least 7 |
| 9. How many servings of legumes do you consume per week? (1 plate or 150 gram serving) | <3 | At least 3 |
| 10. How many servings of seafood do you consume per week? (1 plate or portion: 100-150 grams or 4-5 pieces or 200 grams of seafood) | <3 | At least 3 |
| 11. How many times do you consume commercial (not homemade) pastries such as cookies, custard, candy, or cakes per week? | <2 | At least 2 |
| 12. How many times do you consume nuts and dried fruits per week? (30 grams portion) | <3 | At least 3 |
| 13. Do you preferably consume chicken, turkey or rabbit meat instead of beef, pork, hamburgers or sausages? | Yes | No |
| 14. How many times a week do you eat cooked vegetables, pasta, rice, or other dishes dressed with tomato, garlic, onion or leek sauce simmered with olive oil (sofrito)? | <2 | At least 2 |
| **Do you take folic acid as nutritional supplement?** |  |  |
| Yes, daily |  |  |
| Occasionally |  |  |
| No |  |  |
