# Supplementary figures and images for: Endometrial whole metabolome profile at the receptive phase: influence of Mediterranean Diet and infertility
Source: Front Endocrinol (Lausanne). 2023 Apr 19;14:1120988. doi: 10.3389/fendo.2023.1120988 (PMC10155813; doi:10.3389/fendo.2023.1120988)

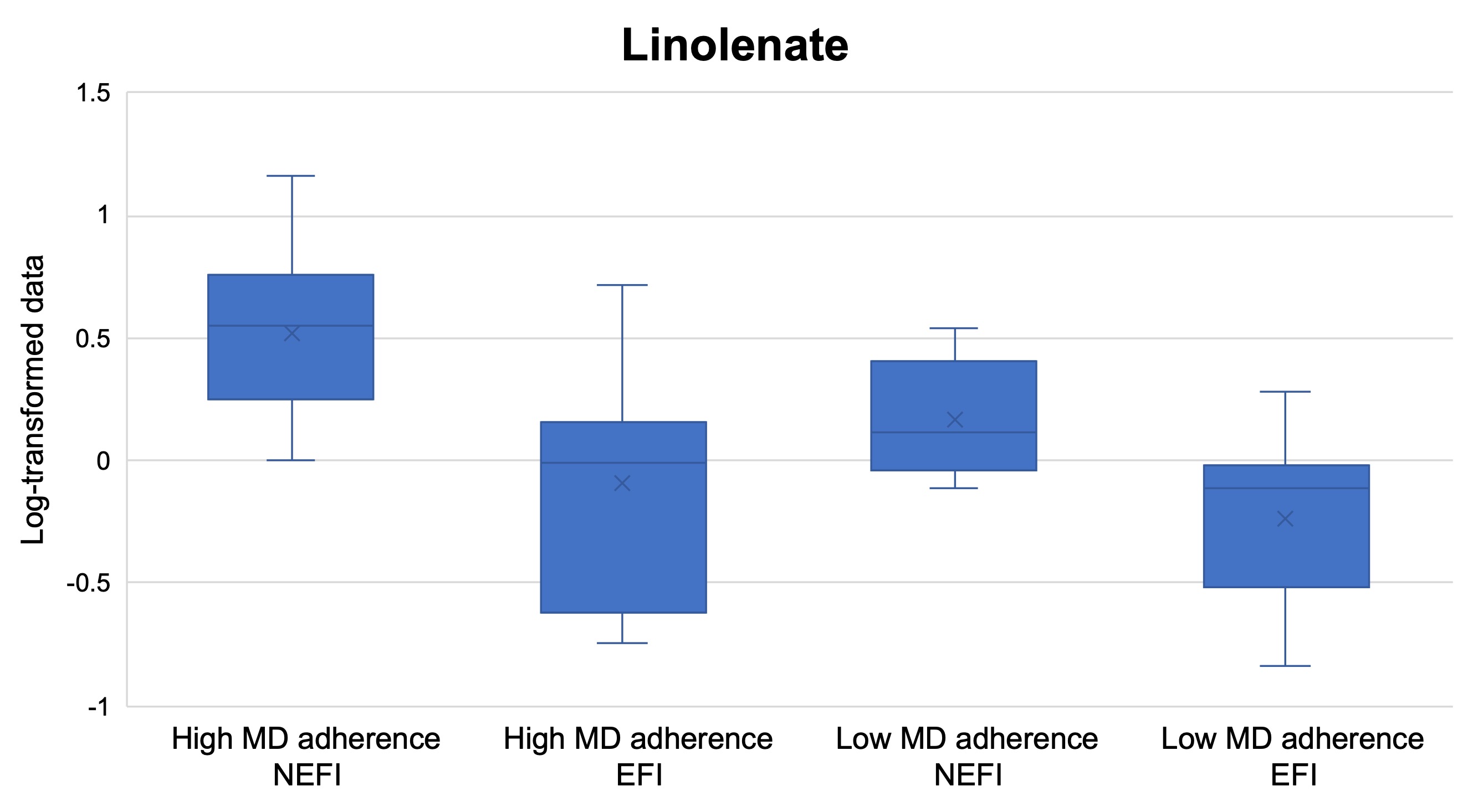

Supplement: Supplementary Figure 1 — Statistical difference in linolenate metabolite between women with high Mediterranean Diet (MD) adherence and no endometrial-factor infertility and women with low MD adherence and endometrial-factor infertility. Statistical significance was set p-value<0.05 after FDR correction. EFI, endometrial-factor infertility; NEFI, no endometrial-factor infertility. [file Image_1.jpg]
